# Mechanical and manual cardiopulmonary resuscitation in traumatic cardiac arrest: a retrospective observational study

**DOI:** 10.1007/s00068-026-03233-1

**Published:** 2026-06-16

**Authors:** Ayman El-Menyar, Khalid Ahmed, Ian Ronald Howland, Monira Mollazehi, Ahammed Mekkodathil, Sandro Rizoli, Peter Cameron, Airton Leonardo de Oliveira Manoel, Hassan Al-Thani

**Affiliations:** 1 Trauma Surgery, Clinical Research, Hamad Medical Corporation, Doha, Qatar; 2 Clinical Medicine, Weill Cornell Medicine, Doha, Qatar; 3 Trauma Surgery, Hamad Medical Corporation, Doha, Qatar; 4 Emergency Medical Services (EMS), Hamad Medical Corporation, Doha, Qatar; 5 Department of Emergency, Hamad Medical Corporation, Doha, Qatar

**Keywords:** Traumatic cardiac arrest, Prehospital care, Mechanical CPR, Manual CPR, LUCAS^®^ device, Bystander CPR, Cardiac rhythm

## Abstract

**Background:**

Prehospital Traumatic cardiac arrest has a high fatality rate despite advances in trauma systems and resuscitation strategies. Mechanical chest compression devices have been increasingly adopted to optimize cardiopulmonary resuscitation (CPR) during Emergency Medical Services (EMS) transportation. We aimed to evaluate the impact of CPR (mechanical during transportation versus manual CPR only at the scene) on survival among trauma patients.

**Methods:**

A retrospective analysis was conducted for patients who received CPR at the scene and during transportation to the hospital between 2016 and 2024.

**Results:**

A total of 610 patients sustaining blunt traumatic cardiac arrest were included. The mean age was 34 ± 12.6 years; 94.6% were male, and 5.4% were female patients. Two hundred twenty (36.1%) received mechanical CPR during transportation to the hospital, and 390 (63.9%) received manual CPR only at the scene. Compared with the manual CPR group, the mechanical CPR group had higher rates of primary chest injury (30.5% vs. 20.5%; *p* = 0.006), bystander CPR (15.9% vs. 6.7%; *p* = 0.001), adrenaline administrations (95% vs. 26.4%; *p* = 0.001), and initial non-shockable rhythm (80% vs. 27.2%; *p* = 0.001). The two groups were comparable in median Injury Severity Score (ISS), head Abbreviated Injury Score (AIS), and total transport time. Mechanical CPR was associated with a markedly higher survival to hospital arrival (98.6% vs. 38.5%, *p* = 0.001), however, the rate of return of spontaneous circulation (ROSC) on arrival to the hospital remained low in both groups (10.5% with mechanical CPR vs. 5.9% with manual CPR, *p* = 0.041), and the overall 30-day survival rate for the entire cohort was just 0.7% (*n* = 4/610). The first 24-hour in-hospital survival rate was significantly higher in the mechanical CPR group than in the manual CPR group (93.1% vs. 89.3%). Among survivors to hospital arrival, the 30-day survival rate was 1.1% (*n* = 4/367) overall, 2.7% (*n* = 4/150), in the manual CPR group, and 0% (*n* = 0/217) in the mechanical CPR group. Age-gender-adjusted predictors for ROSC at handover were ISS (aOR 0.97; 95% confidence interval (CI) 0.95–0.99, *p* = 0.03) and thoracentesis (aOR 3.9; 95% CI 1.50–9.98, *p* = 0.005).

**Conclusions:**

Mechanical CPR in prehospital blunt traumatic cardiac arrest was associated with improved survival to hospital arrival compared with manual CPR; however, in-hospital mortality remained extremely high. These findings suggest that mechanical CPR may facilitate transport of patients in traumatic cardiac arrest without substantially improving overall outcomes. While mechanical devices can provide continuous chest compressions during transport, their impact on meaningful survival in the trauma setting appears limited. The effect of potential iatrogenic injuries associated with the CPR process itself was lacking in this study. Further studies are needed to clarify the role and indication of prehospital mechanical CPR in traumatic cardiac arrest.

**Clinical trial number:**

Not applicable for this retrospective study.

## Background

Cardiopulmonary resuscitation (CPR) is a crucial intervention following traumatic cardiac arrest (TCA). It must be timely, continuous, and effective until return of spontaneous circulation (ROSC) is achieved or resuscitation is terminated [1]. In witnessed TCA with a shockable rhythm, immediate defibrillation is prioritized, followed by high-quality chest compressions. In an unwitnessed cardiac arrest or when defibrillation is not immediately available, chest compressions should be initiated without delay. Manual CPR, the gold standard for resuscitation, may be limited by the inconsistent quality, provider fatigue, and variable outcomes [2, 3].

Mechanical CPR, when available, offers an alternative option by providing automated, uninterrupted compressions during Emergency Medical Services (EMS) transport; however, delayed deployment may compromise early resuscitation quality and reduce survival [4, 5]. Clinical trials and meta-analyses have compared mechanical and manual CPR in non-traumatic settings, whereas trauma-specific evidence remains limited. Notably, the underlying pathophysiology and causes of cardiac arrest in trauma differ substantially from non-traumatic arrest, meaning that evidence from medical cardiac arrest does not necessarily apply to TCA patients. An umbrella review suggested that mechanical CPR may be inferior to manual CPR overall but may be beneficial in selected situations [4–8]. Device-related complications, including rib fractures, cardiac injury, and liver injury, have also been reported in mechanical CPR [9].

Current resuscitation guidelines emphasize the delivery of consistent, high-quality chest compressions with adequate depth, rate, and recoil, whether performed manually or with mechanical devices [10]. Mechanical CPR systems are used in prehospital and in-hospital settings due to their portability and automated compressions and are broadly categorized into load-distributing band devices (e.g., AutoPulse, Zoll) and piston-driven devices (e.g., LUCAS^®^, Stryker) [11]. By delivering standardized compression, mechanical CPR may reduce variability associated with manual compressions and mitigate provider fatigue [12, 13]. This consistency may help maintain perfusion during prolonged resuscitation or patient transport, situations in which effective manual CPR can be difficult to sustain, particularly in remote out-of-hospital cardiac arrest and trauma settings [10, 12, 13]. Mechanical CPR has therefore been considered, especially in certain situations.

Many Studies on mechanical CPR devices for cardiac arrest have shown a little survival benefit in non-trauma settings, whereas there are few reports of mechanical CPR devices for TCA [4, 8, 10]. Experimental and clinical observations suggest that these devices may improve coronary and cerebral perfusion compared with manual CPR during extended resuscitation efforts [10, 12, 14, 15]. In TCA, where injuries may limit access to the chest that require ongoing interventions, maintaining continuous compression is rendered challenging [16–21]. However, studies evaluating mechanical CPR in ED settings have also reported device-related interruptions and logistical challenges that may negatively affect outcomes [22–24]. Given these mixed findings and the limited trauma-specific evidence, the present study aimed to evaluate the impact of mechanical CPR in patients with TCA compared with manual CPR alone.

## Methods

In this retrospective study, data were extracted from medical records and the Qatar National Trauma Registry (QNTR) at the Level I Trauma Center, with approval from the medical research center. This registry prospectively collects data from the Hamad Trauma Center and reports them to the American College of Surgeons National Trauma Data Bank (NTDB) and Trauma Quality Improvement Program (TQIP). The registry undergoes regular (quarterly) internal and external validation as a part of the NTDB [25].

### Objectives

We aimed to investigate whether mechanical CPR during ambulance transportation (within a medically equipped vehicle) following manual CPR (at the scene) impacted the outcome of trauma patients compared with only manual CPR at the scene. We hypothesized that the use of mechanical CPR during prehospital transport would be associated with a higher survival rate compared to manual CPR alone.

### Inclusion criteria (as per the EMS routine protocol)

The study included patients aged ≥ 14 years with prehospital blunt TCA who received CPR and were transported to the hospital by EMS between January 2016 and December 2024. Patients eligible for mechanical CPR (fit to use the device) should have a sternum height of 6.7 to 11.9 inches / 17 to 30.3 cm, and a maximum chest width of 17.7 inches / 45 cm.

### Exclusion criteria

Patients declared dead at the scene, those with unsurvivable injuries without attempted resuscitation, pediatric patients aged < 14 years, and cases with missing prehospital CPR data. Using a CPR device is not restricted by patient weight, but patients who are too small or too large (obese) and do not fit the CPR device are excluded from the use of the mechanical device. Otherwise, following initial manual CPR, patients with continuous cardiac arrest or re-arrest during transportation will receive mechanical CPR until arrival at the HTC.

### Study variables

Collected variables included demographic characteristics (age, sex, and comorbidities), mechanism of injury (MOI), injured body regions, Glasgow Coma Scale (GCS), Injury Severity Score (ISS), Abbreviated Injury Scale (AIS), prehospital time intervals (scene time, response time and total EMS time) [26], CPR at the scene, CPR during EMS transport, initial cardiac rhythm at hospital handover, adrenaline administration (intravenous or intraosseous), endotracheal intubation, activation of Massive Transfusion Protocol (MTP), and survival outcomes.

### Study groups

**Fig. 1:**
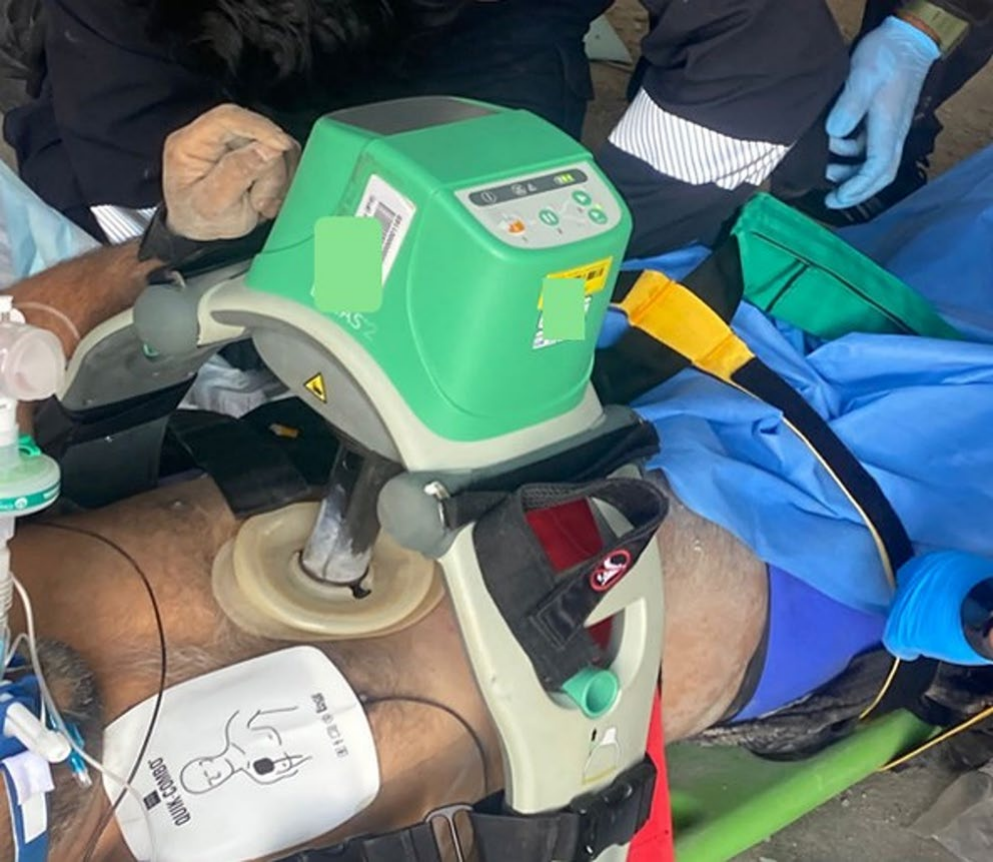
Mechanical CPR during transportation

Patients were categorized into two groups based on the type of chest compressions delivered during transport: the mechanical CPR using the Lund University Cardiopulmonary Assist System (LUCAS^®^) device group (Fig. 1) and the manual chest compressions only group. In all cases, manual CPR was initiated at the scene, and the mechanical device was applied during transport as per the attending critical care paramedics (CCP).

### Study definitions

**Fig. 2:**
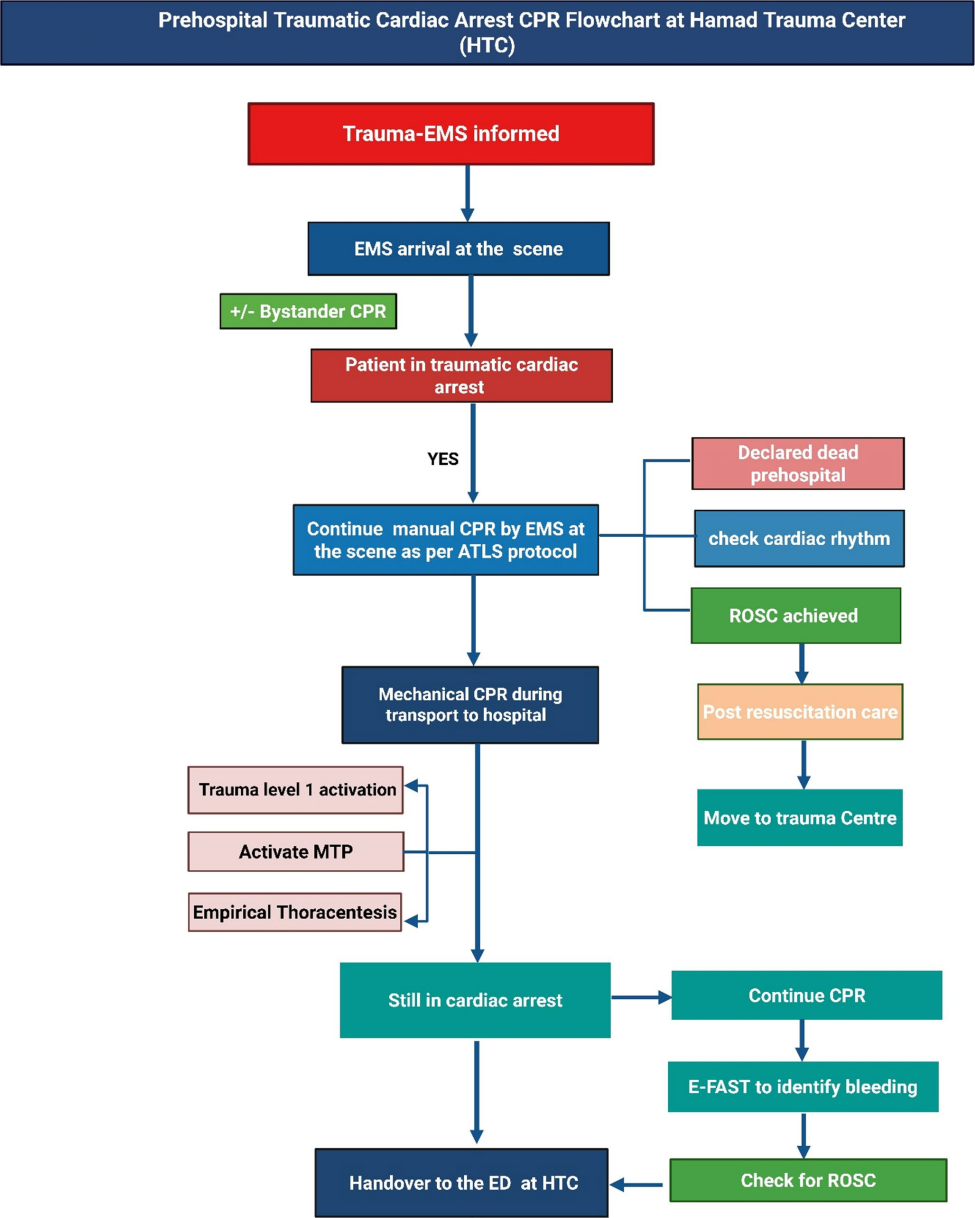
Prehospital traumatic cardiac arrest CPR chart. CPR: cardiopulmonary resuscitation

- ***Age***: The age limit in this study was defined according to the Hamad Trauma Center (HTC) policy, which receives patients of 14 years and above, whereas patients under 14 years are transported directly to pediatric facilities to be managed by the pediatrician team, as there is an agreement across the healthcare sectors in the country in this regard.
- ***AIS*** is defined as 0 (no injury),1 (minor), 2 (moderate), 3 (severe), 4 (severe and life-threatening), 5 (severe and critical, survival uncertain), 6 (maximal, possibly fatal).
- ISS is the sum of squares for the highest AIS grades in the three most severely injured ISS body regions.
- ***ISS*** is defined as mild (ISS 1–8), moderate (ISS 9–15), severe (ISS 16–24), and lethal (ISS ≥ 25) [27].
- ***Study Outcomes***: The primary outcome was survival to hospital arrival, in-hospital survival, and 30-day survival. Secondary outcomes included ROSC on hospital arrival.
- ***Prehospital death*** is defined as the death of a patient with TCA undergoing CPR at the scene or during transport. The EMS team declares the patient dead in the prehospital settings.
- **EMS in Qatar**: The sole EMS in Qatar provides a two-tier service, comprising ambulance paramedics and a CCP team, both trained and certified in trauma resuscitation. The CCP provides advanced prehospital life support to high-acuity patients. The EMS in Qatar deploys seven ground-based and two helicopter critical care teams per shift to respond to the highest-priority emergencies across the country. All cardiac arrests will initiate an automatic dispatch of a CCP in addition to multiple ambulance paramedics. EMS follows the Advanced Life Support (ALS) protocols to guide CPR and interventions. While these interventions are ongoing, EMS plans to transfer to the hospital, ensuring that CPR continues uninterrupted during transport.
- ***CPR***: The timing process is tracked from the moment EMS is notified of a traumatic injury until hospital handover. Upon receiving the call (999 is the sole national emergency number for accessing emergency services in the country), EMS responds and moves to the scene. After confirmation of TCA, manual CPR is initiated immediately. The management of TCA follows a structured approach to address reversible causes while providing high-quality CPR. Figure 2 outlines the key steps in the prehospital management of TCA at the HTC. Mechanical CPR devices are commonly deployed during transport to maintain continuous chest compression when manual CPR is difficult to sustain. Patients are continuously monitored during transport for ROSC.

- ***ROSC*** is the return of a palpable central pulse with blood pressure and with or without spontaneous breathing, together with an increase in the End-Tidal carbon dioxide (CO2). On the way to the hospital, the CCP staff monitors the patient for ROSC. If ROSC is achieved, post-resuscitation care is initiated, focusing on stabilizing the patient and preparing for hospital handover. If ROSC is not achieved, CPR continues until hospital arrival. If no ROSC is achieved and further interventions are deemed futile, the CCP and the receiving team at the HTC declare the patient death.
- **Follow-up in the Trauma Room**: On arrival at the hospital, an ATLS approach with thorough clinical and diagnostic evaluation to identify injury severity and hemodynamic status, along with the appropriate treatment plan.

### Statistical analysis

Continuous variables were summarized as means ± standard deviations (SD) or medians with interquartile ranges, as appropriate, and categorical variables as frequencies and percentages. Patients were categorized into two groups according to the type of chest compressions delivered during transport: mechanical CPR vs. manual chest compressions alone at the scene. Also, to compare no ROSC group vs. ROSC group on arrival. Continuous variables were compared using the Student’s t-test, and categorical variables were compared using the Pearson chi-square (χ²) test. Multivariable regression analysis was performed to determine predictors of ROSC at handover, and results were expressed as age- and gender-adjusted odds ratios (aORs) with 95% confidence intervals (CIs). Variables were used if statistically significant in the univariate comparison model. A two-tailed p-value < 0.05 was considered statistically significant. Statistical analyses were performed using the Statistical Package for the Social Sciences (SPSS Inc., Chicago, IL, USA), version 28. This study was conducted and reported in accordance with the Strengthening the Reporting of Observational Studies in Epidemiology (STROBE) guidelines (Supplementary Material 1).

## Results

**Fig. 3:**
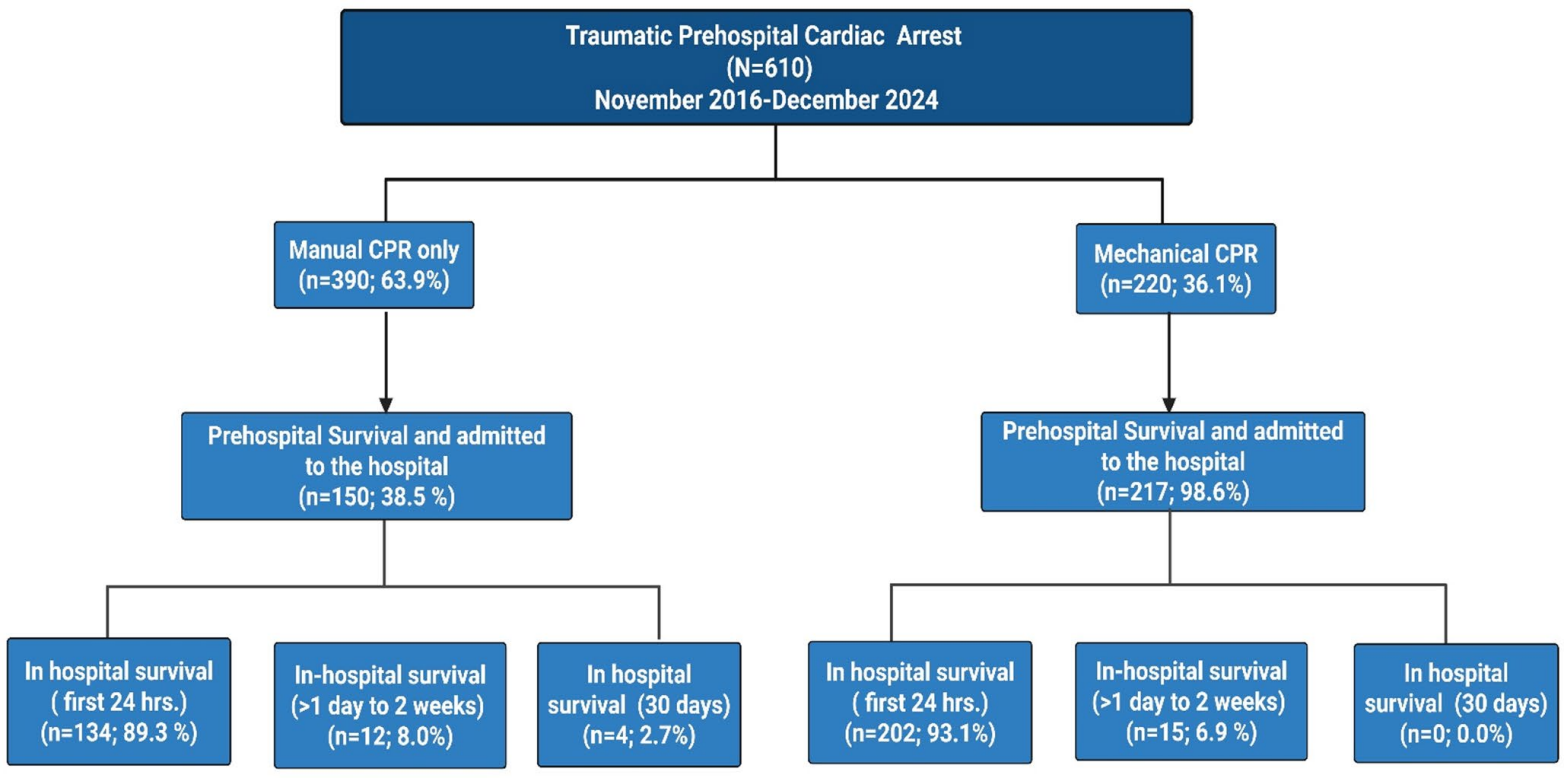
Flow chart of study design. EMS: emergency medical services; CPR: cardiopulmonary resuscitation; ATLS: Advanced Trauma Life Support; MTP: massive blood transfusion protocol activation; E-FAST: extended focused assessment with sonography in trauma; ED: emergency department; ROSC: return of spontaneous circulation

Over the study period, there were 15,750 trauma-related admissions, nearly 90% of which were due to blunt trauma. A total of 610 patients with blunt TCA were transported from the scene to the emergency department at the HTC after CPR. Of these, 390 (63.9%) received manual CPR at the scene, and 220 (36.1%) received mechanical CPR during transportation to the hospital (Fig. 3).

The cohort mean age was 34 ± 12.6 years, 29 patients were aged 14–18 years, 94.6% of patients were male, and 5.4% were female. Six hundred and six patients (99.3%) had blunt trauma, and four had penetrating injuries. Comorbidities included 5 diabetics, three hypertensive and two cardiac patients. Most patients had a head injury (42.3%) with a head AIS score of 4.68 ± 1.1, while primary chest injury was present in 24.1% of patients with a chest AIS score of 3.32 ± 1.34. The overall ISS was 39.78 ± 22.2. The median EMS response time was 8 min (interquartile range [IQR] 5–11), while the on-scene time was 40 min (IQR 29–60), and the total prehospital time was 83 min (IQR 61–114) (Table 1).

**Table 1:** Clinical characteristics of trauma patients with prehospital cardiac arrest by mechanical device use

	Overall (*N* = 610)	Manual CPR (*n* = 390, 63.9%)**	Mechanical CPR (*n* = 220, 36.1%)	*P*-value
Mean Age in years	33.6±12.6	35.1±12.6	36.4±12.5	0.225
Males	577 (94.6%)	378 (96.9%)	199 (90.5%)	0.001
Mechanism of injury				
• Road Traffic Accidents	430 (70.5%)	280 (71.8%)	150 (68.2%)	0.175
• Fall	57 (9.3%)	30 (7.7%)	27 (12.3%)	
• Other	123 (20.2%)	80 (20.5%)	43 (19.5%)	
Injured body region				
• Head	258 (42.3%)	164 (42.1%)	94 (42.7%)	0.871
• Head AIS	4.68 ± 1.09	4.67 ± 0.88	4.70 ± 1.47	0.907
• Chest (primary chest injury)*	147 (24.1%)	80 (20.5%)	67 (30.5%)	0.006
• Chest AIS	3.32 ± 1.34	3.24 ± 1.35	3.43 ± 1.34	0.576
Injury Severity Score	29 (25–75)	26 (25–50)	30(25–75)	0.120
Chest AIS ≤ 2	17 (27.4%)	11 (32.4%)	6 (21.4%)	0.336
Chest AIS > 2	45 (72.6%)	23 (67.6%)	22 (78.6%)	0.336
Response time in min (IQR)	8 (5–11)	8 (5–12)	9(6–11)	0.40
Scene time in min	40 (29–60)	39 (27–57)	42 (31–65)	0.80
Total transport time in minutes	83 (61–114)	84 (61–115)	80 (60–109)	0.90
Scene Glasgow Coma Score	3.25 ± 1.46	3.13 ± 0.98	3.40 ± 1.89	0.581
FAST positive	23 (3.8%)	5 (1.3%)	18 (8.2%)	0.001
Thoracentesis	97(15.9%)	38 (9.7%)	59 (26.8%)	0.001

AIS: abbreviated injury scale. * Chest injury due to accident or fall and not related to the CPR complication. FAST: Focused Assessment with Sonography in Trauma

### Mechanical versus manual CPR

Compared with the manual CPR group, patients who received mechanical CPR had higher rates of primary chest injury (30.5% vs. 20.5%, p = 0.006), bystander CPR (15.9% vs. 6.7%; p = 0.001), continuation of CPR during transport (69.8% vs. 16.3%, p = 0.001), adrenaline administration (95% vs. 26.4%; p = 0.001), non-shockable pulseless electrical activity (PEA) at presentation (41% vs. 19.6%; p = 0.001), and shockable VF/VT (10.5% vs. 0.6%; p = 0.001) (Table 2).

**Table 2:** Cardiopulmonary resuscitation (CPR) and Outcomes of patients with traumatic prehospital cardiac arrest

	Overall (*N* = 610)	Manual CPR only (*n* = 390, 63.9%)	Mechanical CPR (*n* = 220, 36.1%)	*P*-value
EMS witnessed cardiac arrest	45 (11.5%)	15 (8.5%)	30 (14.0%)	0.087
Bystander CPR done	61 (10.0%)	26 (6.7%)	35 (15.9%)	0.001
First recorded rhythm				
• Non-shockable asystole	243 (64.5%)	125 (76.7%)	118 (55.1%)	0.001
• Non-shockable PEA	120 (31.8%)	32 (19.6%)	88 (41.1%)	
• Non-shockable narrow complex organized with pulse	12 (3.2%)	5 (3.1%)	7 (3.3%)	
• Shockable VF/VT	2 (0.5%)	1 (0.6%)	1 (10.5%)	
Rhythm at the time of transportation				
• Non-shockable asystole	161 (55.3%)	78 (72.2%)	83 (45.4%)	0.001
• Non-shockable PEA	85 (29.2%)	14 (13.0%)	71 (38.8%)	
• Non-shockable narrow complex organized	38 (13.1%)	16 (14.8%)	22 (12.0%)	
• Shockable VF/VT	7 (2.4%)	0%	7 (3.8%)	
Rhythm at ED handover				
• Non-shockable asystole	191 (67.5%)	77 (75.5%)	114 (63.0%)	0.001
• Non-shockable PEA	50 (17.7%)	7 (6.9%)	43 (23.8%)	
• Non-shockable narrow complex organized	39 (13.8%)	18 (17.6%)	21 (11.6%)	
• Shockable VF/VT	3 (1.1%)	0%	3 (1.7%)	
Adrenaline	312 (51.1%)	103 (26.4%)	209 (95.0%)	0.001
ROSC at handover (ED)	46 (7.5%)	23 (5.9%)	23(10.5%)	0.041
Massive Transfusion	15 (2.5%)	8 (2.1%)	7 (3.2%)	0.387
**Outcome (survival)**				0.001 for all
Prehospital survival	367/610 (60.2%)	150/390 (38.5%)	217/220 (98.6%)	
In-hospital survival (first24 hours)	336/367 (91.6%)	134/150 (89.3%)	202/217 (93.1%)	
In-hospital survival (> 1 day to 2 weeks)	27/367 (7.3%)	12/150 (8%)	15/217 (6.9%)	
30-day survival	4/367 (1.1%)	4/150 (2.7%)	0/217 (0.0%)	0.001

CPR: Cardiopulmonary resuscitation; PEA: Pulseless Electrical Activity; EMS: Emergency Medical Service, ROSC: return of spontaneous circulation

### ROSC

The overall ROSC at hospital arrival was 7.5% (46/610). At handover, ROSC was higher in patients with lower ISS and underwent more thoracentesis than in the no-ROSC group. (Table 3). Multivariable regression analysis showed that thoracentesis (aOR 3.9; 95% CI 1.50–9.98, p = 0.005) was an age and gender-adjusted predictor of ROSC at handover.

**Table 3:** Return of spontaneous circulation at handover on arrival to hospital

	No ROSC (*n* = 321)	ROSC (*n* = 46)	*P*-value
Age in years	35.9±12.4	34.1±12.6	0.33
Male	301 (93.8%)	43 (93.5%)	0.73
Injury Severity Score (ISS)	46.2 ± 24.7	33.8 ± 11.1	0.005
Mechanical CPR used	194 (60.4%)	23 (50.0%)	0.18
Initial ECG rhythm (non-shockable)	301 (99.3%)	40 (100%)	0.61
Adrenaline given	274 (85.4%)	35 (76.1%)	0.11
Bystander CPR	51 (15.9%)	4 (8.7%)	0.21
Thoracentesis	76 (23.7%)	17 (37.0%)	0.05
Positive FAST	17 (19.5%)	5 (13.9%)	0.45
30-day survival	0 (0%)	4 (8.7%)	0.001

ECG: electrocardiogram, FAST: focused assessment with sonography in trauma

### CPR in children and females (subgroups)

Table 4 shows the clinical characteristics and outcomes of two subgroups: females and children aged 14–18 years old with prehospital TCA. Mechanical CPR was used in 63.6% of females and in 38.7 of the pediatric group. Almost one-third of each group died before arrival at the hospital. The 30-day survival was 0/33 in females and 1/29 (3.4%) in pediatric patients.

**Table 4:** Description of clinical characteristics and outcomes of female patients and children (14–18 years) with prehospital traumatic cardiac arrest

	Females (*n* = 33)	Children (*n* = 29)
Mean Age	37.0±16.5	14.2 ±5.5y
Males	-	27 (93.1%)
Road Traffic Accidents	22 (66.7%)	23 (79.3%)
Fall	3 (9.1%)	2 (6.9%)
Head injury	14 (42.4%)	12 (41.4%)
Primary Chest injury	8 (24.2%)	6 (20.7%)
Injury Severity Score	26.0 (25.0–75.0)	35.5 (25–75)
Scene Glasgow Coma Score	3 (3–3)	3 (3–3)
EMS witnessed cardiac arrest	3 (9.1%)	1 (3.4%)
Bystander CPR done	6 (18.2%)	6 (20.7%)
Mechanical CPR used	21 (63.6%)	12 (41.4%)
Adrenaline	24 (72.7%)	17 (58.6%)
ROSC at handover (ED)	3 (9.1%)	2 (6.9%)
Prehospital survival	23 (69.7%)	18 (62.1%)
• In-hospital survival 0–24 H	22 (95.7%)	17 (94.4%)
• In-hospital survival > 24 H-2 weeks	1 (4.3%)	0 (0%)
• 30-day survival	0 (0%)	1 (3.4%)

CPR: Cardiopulmonary resuscitation; ROSC: return of spontaneous circulation; ED: emergency department, H: hours

### Outcome (Survival)

The overall 30-day survival rate was 0.7% (n = 4/610). Among prehospital survivors, the 30-day survival was 1.1% (n = 4/367) overall, 2.7% (n = 4/150) in the manual CPR group and 0% (n = 0/217) in the mechanical CPR group (Table 5).

**Table 5:** Demographic, presentation and outcome of the survived to discharge cases (all received manual CPR and no mechanical CPR; *n* = 4)

Age (years)	Sex	MOI	ISS	Transferred to Rehabilitation	Neurological Status at Discharge	Follow-up (years)
17	Male	MVC	33	Yes	After 45 days. Conscious, oriented to time and place, no cognitive deficit	Seven
20	Male	MVC	22	No	After 80 days. No significant cognitive impairment affecting performance or self-care	Seven
30	Male	Electric shock & fall from 6 m height	34	Yes	After 57 days. Conscious, not oriented, obeys simple commands	Six
34	Male	MVC	5	No	After 9 days. Conscious, alert, and oriented	Two

MVC: Motor vehicle crash, ISS: Injury severity score; MOI: Mechanism of Injury

### Survivors’ follow-up

Table 5 presents the demographics, presentations, and outcomes of the 4 surviving patients at hospital discharge. All were young males (17–34 years old), received manual CPR only, and had a follow-up between 2 and 7 years. Three out of four had no neurological impairment at discharge, while one patient was conscious but not well oriented.

## Discussion

To the best of our knowledge, studies that have directly compared manual CPR and mechanical CPR exclusively in TCA are scarce, and most meta-analyses excluded trauma-related cardiac arrest patients [6, 10, 28–30]. The present study demonstrated improved survival to hospital admission when mechanical CPR was used during patient transport. However, the survival remained extremely poor (0.7% overall and 1.1% after excluding prehospital CPR deaths), which is lower than what has been reported in a recent meta-analysis (pooled survival of 2.8% including prehospital deaths and 7.7% after excluding prehospital deaths) [31]. Moreover, the pooled survival was 3.4% in studies without a physician-based EMS system, such as our services, compared with 6.1% in studies with a physician-based EMS service [31]. Moreover, our study showed that the 30-day survival varied after excluding prehospital deaths, between 0% with mechanical CPR and 2.7% following manual CPR.

In a retrospective study from the Netherlands, Houwen et al. reported a survival to hospital discharge of 3.9% among prehospital TCA patients, and almost half of them had a good neurological status [32]. Notably, the 30-day survival rate or time-to-death was not explicitly reported in most previous studies. These findings suggest that mechanical CPR may facilitate transport of TCA survivors but does not substantially alter the poor outcomes determined by the severity of traumatic injuries and delivered EMS and ED services.

Most patients in this cohort sustained severe blunt trauma, with high rates of head and chest injuries, elevated ISS, and low GCS values, indicating profound physiological compromise. Patients’ age, MOI, injury severity, and prehospital time intervals were comparable between the two groups, suggesting a similar baseline trauma burden. The evidence of hemorrhagic injuries (i.e., higher rates of positive focused assessment with sonography in trauma (FAST) and thoracentesis) was higher in patients who received mechanical CPR compared with manual CPR in our cohort. Mechanical CPR, compared to manual CPR was performed more in patients with severe chest injury with AIS > 2 (78.6% vs. 67.6%, *p* = 0.33). However, determining which types of chest injuries mechanical CPR devices should be used remains a major challenge at the prehospital scene.

Because effective CPR is difficult in a moving ambulance or flying helicopter, the CPR device was used during transport and continued until arrival at the hospital, where care was transferred to the trauma team, or the patient was declared dead by the EMS team during transport to the hospital. The EMS standard practice is manual chest compressions, with the device reserved for transfer to the hospital. In trauma, mechanical CPR has been shown to provide superior chest compressions compared to manual CPR, particularly in penetrating trauma [16, 17], potentially reducing further injury and improving venous return and cardiac output [22, 23].

In the present study, patients receiving mechanical CPR predominantly presented with non-shockable rhythms at the time of cardiac arrest. Almost all trauma patients with prehospital TCA transported to the trauma resuscitation room (excluding those declared dead before ED arrival or those who achieved ROSC) maintained a non-shockable rhythm until handover to the trauma team. Patients who achieved ROSC at hospital handover were more likely to undergo thoracentesis, have a lower ISS, and have better 30-day survival (3.4%) than those who did not achieve ROSC at handover. However, ROSC at hospital handover was 7.5%, which was lower than the results of Houwen et al. study (28.5%) [32]. Notably, in the Houwen study, the penetrating injury group comprised only 16.5% of the cohort and the survival difference compared to the blunt injury group was not statistically significant (*p* = 0.069). Prior data showed that non-shockable ECG rhythm following TCA is the only patient-related predictor for higher mortality with a Risk Ratio of 1.12 [31]. Our prior data showed that an initial shockable rhythm following TCA was associated with a 6-fold increase in ROSC (adjusted odds ratio 6.39, *p* = 0.02) [33]. The current study revealed that only 7 patients (1.1%) presented with a shockable rhythm.

This present study showed that adrenaline administration differed between the two groups (95% in the mechanical CPR vs. 26% in the manual CPR group). Our prior study (data were collected between 2010 and 2015) revealed that adrenaline use during CPR was associated with lowered survival in the out-of-hospital traumatic cardiac arrest, with an unadjusted odds ratio of ROSC on admission at 0.53 (95% CI 0.3–0.9). The rate of mechanical CPR use in that study was only 6.1% [33].

All patients from the mechanical CPR group died within 2 weeks in the hospital, with the highest number was during the 1st hour. This finding reflects the severity of injuries and underscores the need to better evaluate survival to hospital admission, continued need for resuscitation, and the efficacy of mechanical CPR during transport. Thus, questions remain for future research in trauma patients who remain in TCA during prehospital transport: does earlier or protocol-driven application of mechanical CPR (compared to manual CPR or selective device use) improve rates of survival and quality of life, and which patient or injury characteristics predict benefit from mechanical CPR. Addressing this question through well-designed, prospective randomized trials will help clarify the optimal indications and timing for mechanical CPR deployment in TCA. Involving organ donation coordinator is also advisable for family approach and follow-up.

### Limitations

The main limitation of this study is its retrospective design, which is subject to selection bias and unmeasured confounders. Device-related complications and autopsy findings were not documented, limiting assessment of potential harm from either manual or mechanical CPR; prior studies have reported increased risks of rib fractures, cardiac injury, and liver injury with mechanical CPR [9, 34]. Of note, mechanical CPR devices were used regardless of the type of chest trauma as the latter cannot be assumed without imaging support in the prehospital setting. Therefore, it could be possible that cases with a potential risk of injury from mechanical CPR were included. Prior reports showed that CPR is associated with iatrogenic injury even in non-trauma patients [9, 34]. Our registry did not capture such iatrogenic injuries, therefore along with the lack of autopsy information, we cannot assume the contribution of these injuries to the mortality following CPR. In general, CPR itself may be associated with life-threatening injuries, however data on these iatrogenic injuries- related mortality are lacking. Preda et al. reported life-threatening injuries of 11.7% in their entire cohort, 29.2% following mechanical CPR and 4.9% following manual CPR [9]. Koster et al. reported 7.4–11.7% following mechanical CPR and 6.3% following manual CPR [35]. However, traumatic cardiac arrest patients were not included in these two studies. Miller et al. in a systematic review reported hemopericardium in 8.5% of manual CPR and 7.9% following mechanical CPR in non-traumatic cardiac arrest patients [36]. Additionally, data on emergency thoracotomy or empirical chest tube insertion were not fully obtained. The needle thoracentesis is part of the CPR process in our EMS service. Finger thoracostomy was introduced at the end of May 2024; therefore, no such data were captured for this study.

Survival bias cannot be ignored. Patients who achieved ROSC and thus survived the prehospital phase after manual CPR may differ substantially from those continuing to require CPR during transport, who were more likely to receive mechanical CPR and often had more severe or non-reversible injuries.

Moreover, the duration of resuscitation when prehospital ROSC was achieved, and the time between the manual CPR and the application of the CPR device were not documented in the patient’s chart. Due to the design of this study, it was not possible to track this information. Moreover, the results could be sex-biased as 94% were male; however, it reflects the nature of population characteristics of trauma in Qatar [37].The 30-day survival was 0% among the 33 female patients in the study cohort, of whom almost two-thirds received mechanical CPR.

The actual number of prehospital TCA is underestimated, as victims who were found dead at the scene were not captured in this analysis, not counted in the trauma registry, and were transferred directly to the mortuary. Moreover, the impact of the whole body size on the choice of the type of CPR was not explicitly described.

## Conclusion

Mechanical CPR was associated with improved survival to hospital arrival in blunt TCA but did not improve overall survival, which remained extremely low. These findings suggest that mechanical CPR may facilitate transport of patients in arrest without substantially altering outcomes determined by the severity of traumatic injuries. While mechanical devices can provide continuous chest compressions during transport, their impact on meaningful survival in trauma patients appears limited. The effect of potential iatrogenic injuries associated with the CPR process itself was lacking in this study. Further prospective studies are needed to better define the role of mechanical CPR in TCA and to identify clinical scenarios in which its use may be beneficial.

## Data Availability

All data are presented in the manuscript. It will be available at reasonable request and approval from the medical research center of Hamad Medical Corporation.
